# [Corrigendum] Src homology phosphotyrosyl phosphatase 2 mediates cisplatin-related drug resistance by inhibiting apoptosis and activating the Ras/PI3K/Akt1/survivin pathway in lung cancer cells

**DOI:** 10.3892/or.2025.9026

**Published:** 2025-11-19

**Authors:** Chunlan Tang, Hu Luo, Dan Luo, Heping Yang, Xiangdong Zhou

Oncol Rep 39: 611–618, 2018; DOI: 10.3892/or.2017.6109

Following the publication of this paper, and the publication of an Expression of Concern statement (doi.org/10.3892/or.2025.8979), the authors have replied concerning an issue that was drawn to our attention by an interested reader; namely, that the immunohistochemical images shown in Figs. 1E and 5E appeared to show an overlapping section, even though Figs. 1 and 5 were intended to show the results of SHP2 and Ras expression experiments, respectively.

The authors were able to check their data, and realized that Fig. 5 had inadvertently been assembled incorrectly. The revised version of Fig. 5, now showing the correct data for Fig. 5E, is shown below. Note that these errors did not adversely affect either the results or the overall conclusions reported in this study. All the authors agree with the publication of this corrigendum, and are grateful to the Editor of *Oncology* *Reports* for allowing them the opportunity to publish this. They also wish to apologize to the readership of the Journal for any inconvenience caused.

## Figures and Tables

**Figure 5.**
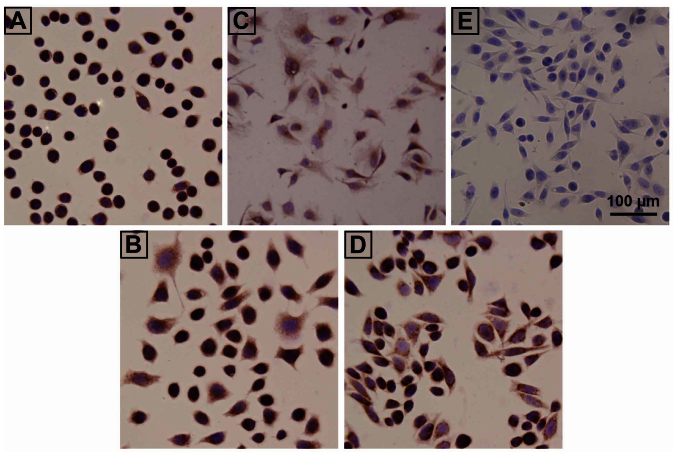
Ras expression in cisplatin-induced drug-resistant and parental cells. The Ras expression was determined by immunohistochemistry in (A) SPC-A-1, (B) SPC-A-1/CDDP, (C) H446, (D) H446/CDDP and (E) negative control, H446 cells incubated with primary antibody diluents without primary antibody (magnification, ×400). CDDP, cisplatin.

